# The Danger of Smoking on Home Oxygen: A Cautionary Case of Facial Flash Burn

**DOI:** 10.1155/crpu/6623790

**Published:** 2025-11-21

**Authors:** Angela Fadil, Samuel E. Kennedy, Adam Austin

**Affiliations:** Department of Medicine, Division of Pulmonary, Critical Care and Sleep Medicine, University of Florida College of Medicine, Gainesville, Florida, USA

## Abstract

For patients on oxygen therapy, close encounters with open flames carry a risk of morbid burns and airway injuries. Despite this, many patients prescribed long-term home oxygen therapy continue to smoke. This article describes one such case, demonstrating the consequences and emphasizing the importance of physician counseling and patient education.

## 1. Introduction

Long-term home oxygen therapy (HOT) remains the standard of care for hypoxemia in end-stage lung disease. Although open flames pose an explicit danger in the proximity of oxygen devices, an estimated 15%–25% of patients prescribed oxygen continue to smoke [1]. Smoking is cited to cause 83% of burn injuries associated with home oxygen use [2]. While burn injuries to the face are a rare occurrence, they carry high morbidity and mortality, especially the notable proportion with concomitant inhalational injury [3]. A review of HOT guidelines suggested a failure to appropriately emphasize the hazards of smoking, indicating a potential deficit in patient education that contributes to the incidence of these morbid injuries [4]. The incidence of smoking-related injuries calls for greater emphasis on patient education and provider judgment in prescribing HOT. This case report illustrates the hazards of smoking on supplemental oxygen therapy, emphasizing the role of patient education and counseling to minimize the risk of burn injury.

## 2. Case Presentation

**Figure 1:**
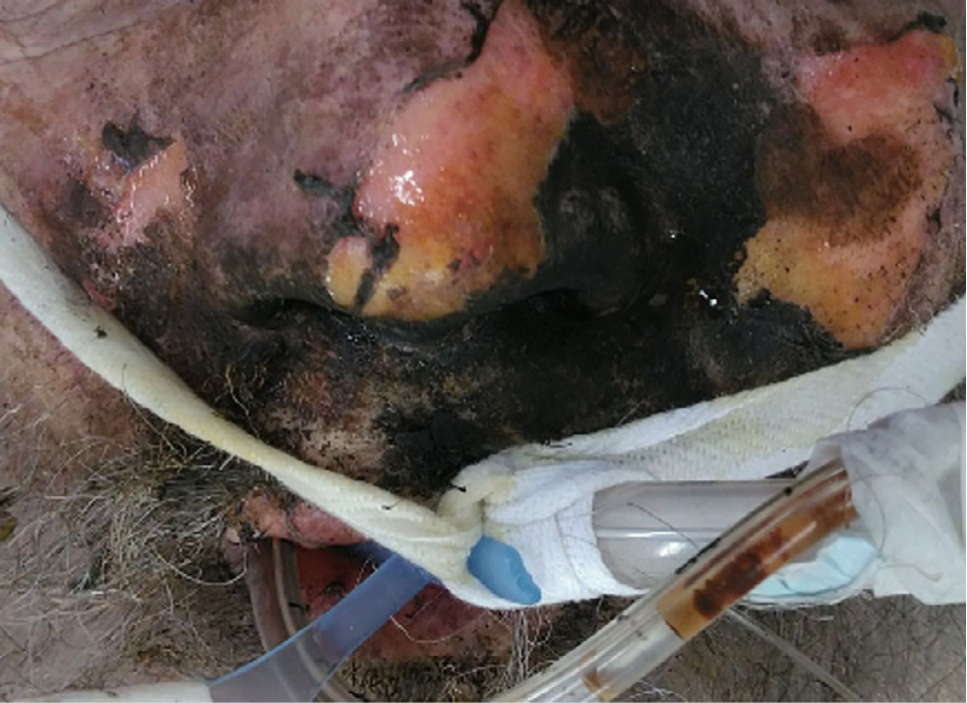
Facial burn. Partial thickness flash burn sustained to the face while smoking a cigarette on home oxygen therapy.

A 67-year-old man with chronic obstructive pulmonary disease and congestive heart failure presented to the emergency department after sustaining a flash burn to the face while smoking a cigarette while using home oxygen. Emergency medical services performed endotracheal intubation on scene for acute respiratory distress. On presentation, he was tachycardic, normotensive, and saturating 99% on ventilatory support. Physical exam was significant for wheezing, partial thickness burns diffusely to the face, soot in the nares and oropharynx, and singed facial hairs (Figure 1).

**Figure 2:**
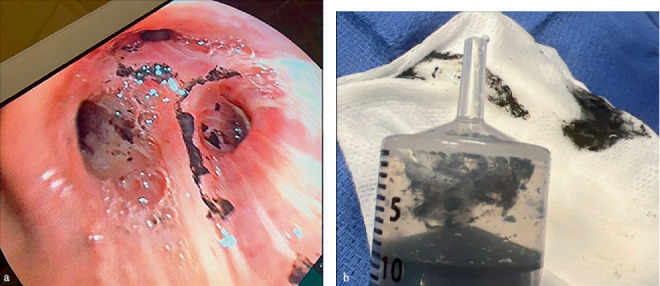
Bronchoscopy. (a) View of the right upper lobe during initial bronchoscopy. (b) Carbonaceous material obtained from bronchoalveolar lavage.

He was admitted to the intensive care unit where bronchoscopy was performed. Bilateral airways had copious soot and mucinous secretions. Views of the airways were predominately Grade 1 with one area of Grade 2 at the distal left main bronchus and left upper lobe. Bronchoalveolar lavage produced dark, particulate fluid (Figure 2). Repeat bronchoscopy with irrigation was performed on Days 1 and 2 following injury, demonstrating progressively improving airway edema and decreased carbonaceous material. Plastic surgery performed initial debridement of the facial burns, which ultimately did not require surgery. The patient was extubated on Day 5 and discharged home on Day 16 with continued HOT. Prior to discharge, he received extensive counseling on tobacco cessation and the dangers of smoking while on oxygen.

## 3. Discussion

Inhalation burns are known to cause significant morbidity and mortality. The mechanism of airway injury is due to both direct thermal and chemical insult. These injuries may involve the supraglottic or subglottic airway, producing swelling and airway obstruction, highlighting the importance of critical airway management in these patients. Delayed airway injury can also lead to complications including pneumonia, tracheomalacia, bronchospasms, and acute respiratory distress syndrome [3]. Additionally, these patients may experience systemic inhalation injury secondary to carbon monoxide or cyanide exposure. The mortality benefits of supplemental oxygen in patients with hypoxemia are well described in the literature [1]. However, given the known risk of burn injury, nations like Sweden even restrict HOT for patients actively smoking [5]. Rather than enact policy change among those countries that do not restrict HOT prescriptions, the authors maintain that patients in need of oxygen therapy who continue to smoke require careful risk–benefit discussions. Clinicians prescribing and managing HOT must appropriately counsel patients and caregivers to mitigate the risks of burn injury. Healthcare providers should specifically inquire about e-cigarettes and nontobacco inhalants, including marijuana, which also pose a risk of flash fire injury. Accurately identifying which patients are still smoking will help appropriately distribute attention and resources. For patients with tobacco dependence, frequent re-evaluations and aid in smoking cessation are essential preventative measures. Considering risk factors like mental health status and substance use allows clinicians to risk stratify patients, identifying those who may require closer compliance monitoring while receiving HOT [6, 7].

## Data Availability

Data sharing is not applicable to this article as no datasets were generated or analyzed during the current study.
